# The interaction between the endocannabinoid system and the renin angiotensin system and its potential implication for COVID-19 infection

**DOI:** 10.1186/s42238-020-00030-4

**Published:** 2020-07-31

**Authors:** Alberto Sainz-Cort, Joost H. Heeroma

**Affiliations:** 1 GH Medical, Carrer d’Osca 4B, Sant Boi de Llobregat, Barcelona Spain; 2 GH Medical, Amsterdam, The Netherlands

**Keywords:** COVID-19, Hypertension, Cannabis, ACE-2, SARS-CoV-2, Endocannabinoid

## Abstract

**Background:**

Coronavirus disease 2019 (COVID-19) is spreading fast all around the world with more than fourteen millions of detected infected cases and more than 600.000 deaths by 20th July 2020. While scientist are working to find a vaccine, current epidemiological data shows that the most common comorbidities for patients with the worst prognosis, hypertension and diabetes, are often treated with angiotensin converting enzyme (ACE) inhibitors and angiotensin receptor blockers (ARBs).

**Body:**

Both ACE inhibitors and ARBs induce overexpression of the angiotensin converting enzyme 2 (ACE-2) receptor, which has been identified as the main receptor used by severe acute respiratory syndrome coronavirus 2 (SARS-CoV-2) to enter into the alveolar cells of the lungs. While cannabinoids are known to reduce hypertension, the studies testing the hypotensive effects of cannabinoids never addressed their effects on ACE-2 receptors. However, some studies have linked the endocannabinoid system (ECS) with the renin angiotensin system (RAS), including a cross-modulation between the cannabinoid receptor 1 (CB1) and angiotensin II levels.

**Conclusion:**

Since there are around 192 million people using cannabis worldwide, we believe that the mechanism underlying the hypotensive properties of cannabinoids should be urgently studied to understand if they can also lead to ACE-2 overexpression as other antihypertensive drugs do.

## Background

As of 20th of July, Coronavirus disease 2019 (COVID-19) reached 14.476.729 cases worldwide, with 605.979 deaths (COVID-19 situation update worldwide, as of 20 July 2020 2020). COVID-19 poor prognosis is related to comorbidities like hypertension, cardiovascular diseases, diabetes mellitus, smoking, chronic obstructive pulmonary disease (COPD), malignancy, chronic kidney disease, obesity and immunocompromising conditions. Among these comorbidities, the prevalence of hypertension is the highest (16.37%) in hospitalized patients diagnosed with COVID-19 infection (Emami et al. 2020). Since cannabinoids regulate the immune system, have anti-inflammatory properties and have an effect on cardiovascular function and hypertension, we believe it is of interest to understand if the use of cannabis could influence COVID-19 symptoms and prognosis. It is important to notice that, as of 16th July, we are not aware of any studies directly testing the effects of cannabinoids on COVID-19 disease progression. However, we can try to understand if cannabinoids would have any effect on the proteins, mechanisms and symptoms that are related to the disease by analyzing published literature. The aim of this article is to describe the latest insights in the potential interaction of the endocannabinoid system (ECS) and the physiological mechanisms of COVID-19 infection, in order to have an idea of how cannabinoids might affect the prognosis of the disease.

## Main text

### RAS and COVID-19 infection

The angiotensin converting enzyme 2 (ACE-2), which belongs to the renin angiotensin system (RAS), has been identified as the main entrance for the severe acute respiratory syndrome coronavirus 2 (SARS-CoV-2) to cause COVID-19 infection in the human body (Fang et al. 2020). The RAS regulates the hemodynamics of the body, it has a very important role in the regulation of blood pressure and it has been related to the pathogenesis of hypertension (Miller and Arnold 2019). Antihypertensive drugs that inhibit either angiotensin converting enzymes (ACE) or angiotensin receptors, have been shown to lead to overexpression of ACE-2 (Fang et al. 2020). The use of ACE inhibitors and angiotensin II receptor blockers (ARBs) has been questioned since overexpression of ACE-2 receptors might result in a worse prognosis of the disease (Fang et al. 2020). However, recent scientific evidence raised the controversy whether ACE-2 receptors are actually downregulated or upregulated by ACE inhibitors and ARBs and whether the use of these drugs form a potential benefit or risk for COVID-19 prognosis (Vaduganathan et al. 2020; Cohen et al. 2020; Curfman 2020; Fosbøl et al. 2020). In fact, a recent retrospective cohort study showed no association between the use of these drugs and the diagnosis or mortality of COVID-19 infection (Fosbøl et al. 2020). Researchers and doctors advise patients who are already taking anti = hypertensive drugs to do not stop the medication. This is because stopping the medication could lead to severe health problems while there is not enough scientific evidence to support a potential risk of its use during COVID-19 disease (Vaduganathan et al. 2020).

### Cannabinoids, hypotension and RAS

The ECS has been linked to cardiovascular modulation in multiple studies, including the modulation of blood pressure (Pacher et al. 2018). In fact, cannabis use and isolated cannabinoid administration (either tetrahydrocannabinol (THC) or cannabidiol (CBD)) is known to induce hypotension in healthy users, among other effects on cardiovascular regulation (Pacher et al. 2018). Hypotensive effects of cannabinoids have been reported to be mediated by the activation of cannabinoid receptor 1 (CB1), which is expressed in vascular smooth muscle and endothelial cells (Pacher et al. 2018). The mechanisms underlying the hypotensive effects of cannabinoids are complex, but animal and human studies point to direct vasorelaxation effects induced by the activation of CB1 and/or to the modulation of vasoactive agonists like angiotensin II (Stanley and O’Sullivan 2014). Not only plant-derived cannabinoids have been linked to hypotension, but also hemp seed oil shows hypotensive effects. Interestingly, these hypotensive effects are mediated by ACE inhibition (Girgih et al. 2014; Orio et al. 2017).

We are not aware of any study linking the ECS to the ACE-2 receptor, but we found some connections between the ECS and the RAS, including some links to the ACE receptor. Cross-talk between the ECS and the RAS has been described in several studies using upregulated RAS rat models, showing that CB1 and angiotensin II type 1 receptor (AT1R) form receptor heteromers with functional interactions and suggesting that hypertensive states are related to lower expression of CB1 and higher levels of angiotensin II (Haspula and Clark 2016; Rozenfeld et al. 2011; Schaich et al. 2016). In line with this and with the effects we previously mentioned concerning cannabis use and hypotension, we found a study examining vascular tissues from rats under different CB1 receptor modulations (activation, blockade and knockout) showing that the activation of CB1 reduces vasoconstrictor and hypertensive effects induced by angiotensin II (Szekeres et al. 2012). Since ARBs are used to reduce hypertension by blocking AT1R and CB1 can modulate AT1R, the hypotensive effects followed by CB1 activation might be induced through similar mechanisms. We also know that these CB1-induced hypotensive effects are related to angiotensin II, which is converted from angiotensin I by ACE receptor (Erdös 1976). Thus, it would be interesting to investigate if CB1-induced hypotension is linked to ARB mechanisms and/or ACE inhibition. If this would be the case, the activation of CB1 by cannabinoids like THC might lead to ACE-2 modulation as it has been showed by other antihypertensive drugs.

It is possible that other cannabinoids which show different interactions with CB1 (i.e. CB1 antagonists) could also lead to ACE-2 modulation. Actually, a recent study demonstrated that high-CBD cannabis extracts can modulate ACE-2 expression in artificial 3D human tissue models, suggesting a therapeutic potential in COVID-19 infection (Wang et al. 2020). However, extracts used in the study had different cannabinoid and terpene profiles, resulting in either upregulation or downregulation of ACE-2 expression. Since the total composition of cannabinoids and terpenes of each extract is unknown, it is not possible to determine which combination of active ingredients can actually downregulate or upregulate ACE-2 expression. In any case, this study would reinforce both the link between ECS and RAS and the idea of a potential effect of cannabis use on ACE-2 receptor expression.

## Conclusion

Although our analysis is based on existing literature and does not involve direct experimental evidence, we speculate that the hypotensive effects of THC and other CB1 agonist cannabinoids might be related to some of the mechanisms of ARBs and/or ACE inhibitors. Since these hypotensive drugs are shown to modulate ACE-2, we believe it is important to investigate whether cannabinoids have the same effect on ACE-2 expression and its potential consequences related to COVID-19 infection. If our speculations are right, using cannabinoids like THC might not be advisable in the context of a potential COVID-19 infection. Some media suggested cannabinoids could be used to treat the cytokine storm related to COVID-19 infection (Sexton 2020). There is actually preclinical evidence suggesting cannabinoids might protect against cytokine storm and they could improve prognosis in the case of sepsis (Dinu et al. 2020). However, we believe such evidence is not enough to recommend the use of cannabinoids in these cases since there are already other drugs available that have been extensively tested to treat such disorders. In case patients decide to continue using cannabis, it would be advisable to avoid the smoking route of administration and aim for other routes of administration.

Since there are around 192 million people using cannabis worldwide (World Drug Report 2020 2020), we believe that the mechanism underlying the hypotensive properties of cannabinoids should be urgently studied to understand if they can also lead to ACE-2 modulation as other antihypertensive drugs do.

## Data Availability

Not applicable.

## Abbreviations

ACE: Angiotensin converting enzyme
ACE-2: Angiotensin converting enzyme 2
ARBs: Angiotensin II type 1 receptor blockers
AT1R: Angiotensin II type 1 receptor
CB1: Cannabinoid receptor 1
CBD: Cannabidiol
COVID-19: Coronavirus disease 2019
ECS: Endocannabinoid system
RAS: Renin angiotensin system
SARS-CoV-2: Severe acute respiratory syndrome coronavirus 2
THC: Tetrahydrocannabinol
